# Functional Pangenome Analysis Shows Key Features of E Protein Are Preserved in SARS and SARS-CoV-2

**DOI:** 10.3389/fcimb.2020.00405

**Published:** 2020-07-27

**Authors:** Intikhab Alam, Allan A. Kamau, Maxat Kulmanov, Łukasz Jaremko, Stefan T. Arold, Arnab Pain, Takashi Gojobori, Carlos M. Duarte

**Affiliations:** ^1^Biological and Environmental Science and Engineering (BESE), Computational Bioscience Research Center (CBRC), King Abdullah University of Science and Technology (KAUST), Thuwal, Saudi Arabia; ^2^Biological and Environmental Science and Engineering (BESE), King Abdullah University of Science and Technology (KAUST), Thuwal, Saudi Arabia; ^3^Centre de Biochimie Structurale, CNRS, INSERM, Université de Montpellier, Montpellier, France; ^4^Research Center for Zoonosis Control, Hokkaido University, Sapporo, Japan

**Keywords:** COVID-19, envelope protein (E), therapeutic targets, ARDS (acute respiratory distress syndrome), SARS-CoV-2, E protein Inhibitors

## Abstract

The spread of the novel coronavirus (SARS-CoV-2) has triggered a global emergency, that demands urgent solutions for detection and therapy to prevent escalating health, social, and economic impacts. The spike protein (S) of this virus enables binding to the human receptor ACE2, and hence presents a prime target for vaccines preventing viral entry into host cells. The S proteins from SARS and SARS-CoV-2 are similar, but structural differences in the receptor binding domain (RBD) preclude the use of SARS-specific neutralizing antibodies to inhibit SARS-CoV-2. Here we used comparative pangenomic analysis of all sequenced reference *Betacoronaviruses*, complemented with functional and structural analyses. This analysis reveals that, among all core gene clusters present in these viruses, the envelope protein E shows a variant cluster shared by SARS and SARS-CoV-2 with two completely-conserved key functional features, namely an ion-channel, and a PDZ-binding motif (PBM). These features play a key role in the activation of the inflammasome causing the acute respiratory distress syndrome, the leading cause of death in SARS and SARS-CoV-2 infections. Together with functional pangenomic analysis, mutation tracking, and previous evidence, on E protein as a determinant of pathogenicity in SARS, we suggest E protein as an alternative therapeutic target to be considered for further studies to reduce complications of SARS-CoV-2 infections in COVID-19.

## Introduction

The World Health Organization declared COVID-19, has been declared a global pandemic by the WHO on March 11th 2020 (https://www.who.int/emergencies/diseases/novel-coronavirus-2019). Its potentially life-threatening consequences also entail serious social and economic repercussions. Three major actions, confinement, detection, and therapy are required to contain this pandemic. Detection and therapy can be guided by virus sequence data, which was first made available in January 2020 at gsaid.org, soon after detection of the first cases. The SARS-CoV-2 genome sequence data are a pivotal resource on its own, but can gain greater value when embedded within those of other *Betacoronavirus*, allowing a comparative pangenomic analysis. This approach can help identify the core genome of *Betacoronaviruses* and extract accessory genomic features shared by a subset of these viruses or unique to SARS-CoV-2. Whereas, core genomic features are required for the virus to be functional, accessory features are candidates to provide insights into the drivers of the unique capacities of SARS-CoV-2 explaining its spread and virulence. Genome annotation and structural modeling can then be used to assess the possible functions of these accessory features and guide approaches to detection and treatment.

Here we apply a comparative pangenomic approach of all *Betacoronavirus* genomes sequenced thus far (Table 1), to detect the core and accessory gene cluster of this genus, and then annotate the functions, further assessed through structural analysis. Furthermore, we suggest that the preservation of key features of the envelope protein, E, between SARS and SARS-CoV-2, plays a central role in the development of the Acute Respiratory Distress Syndrome (ARDS). Analysis of mutations from over 2,000 isolates of SARS-CoV-2 show E protein has no mutations in the key regions. Consequently, we suggest inhibition of E protein can be a good target to alleviate ARDS complication of COVID-19.

**Table 1:** Reference genomes included in this study from taxonomic genus of *Betacoronaviruses*.

**Accession**	**Strain (accession) country**	**Host**	**Taxon**	**Submission date**
AF391541	Bovine coronavirus (AF391541)	Bovine	11128	2002
AY700211	Murine hepatitis virus (AY700211)	Rat	11138	2006
AF029248	Murine hepatitis virus (AF029248)	Rat	11138	2000
AY585228	Human coronavirus OC43 (AY585228) USA	Human	31631	2004
AY597011	Human coronavirus HKUl (AY597011)	Human	290028	2006
EF065505	Bat coronavirus HKU4-1 (EF065505) China	Bat	424359	2007
EF065509	Bat coronavirus HKU5-1 (EF065509) China	Bat	424363	2007
EF065513	Bat coronavirus HKU9-1 (EF065513) China	Bat	424367	2007
FJ938068	Rat coronavirus Parker (FJ938068)	Rat	502102	2009
AY274119	SARS (AY274119) Canada: Toronto	Human	694009	2017
JN874559	Rabbit coronavirus HKU14 (JN874559) China	Rabbit	1160968	2012
JX869059	MERS (JX869059).	Human	1235996	2012
KC164505	MERS (KC164505) United Kingdom	Human	1263720	2013
KF600630	MERS (KF600630) Saudi Arabia	Human	1335626	2014
KC545386	*Betacoronavirus* Hedgehog (KC545386) Germany	Hedgehog	1385427	2014
KC545383	*Betacoronavirus* Hedgehog (KC545383) Germany	Hedgehog	1385427	2014
MG772933	Bat SARS-1ike coronavirus (MG772933) China	Bat	1508227	2018
KF636752	Bat Hp-*betacoronavirus*/Zhejiang2013 (KF636752) China	Bat	1541205	2017
KM349742	*Betacoronavirus* HKU24 (KM349742) China	Rat	1590370	2015
KU762338	Rousettus bat coronavirus (KU762338) China	Bat	1892416	2016
MN988713	SARS-CoV-2 (MN988713) USA	Human	2697049	2020
MN985325	SARS-CoV-2 (MN985325) USA	Human	2697049	2020
MN975262	SARS-CoV-2 (MN975262) China	Human	2697049	2020
MN938384	SARS-CoV-2 (MN938384) China	Human	2697049	2020

*Genomes with same taxon id are highlighted in yellow*.

## Methods

### Collection of *Betacoronavirus* Strains

The NCBI genome assemblies page was searched, on January 26, for taxonid of genus *Betacoronavirus*, resulting in a list of 22 genomes, including four isolates of SARS-CoV-2. We downloaded in addition to this initial list two more assemblies one for a bat coronavirus (MG772933) and another for MERS (KF600630), see Table 1. For easy identification of genes and annotations from GenBank, we included unique locus_tag identifiers containing locus id and an index number. Metadata was collected to define user friendly strain names and to understand phylogenetic trees in the context of virus host, country, taxon id and data release date.

### Creating a Pangenome for *Betacoronavirus*

GenBank annotation and sequences were used as a starting point for this pangenomic analysis study. These GenBank annotations contain heterogeneous identifiers for genes so we included unique locus tags containing the strain accession number and gene index number for computational processing. In addition, we collected metadata for these coronaviruses for displaying on the interactive phylogenetic trees for analysis later on. Gene sequences were compared to each other for sequence similarity, followed by gene clustering. Phylogenetic analysis was carried out on the core gene clusters to produce a species tree and similarly for each gene cluster to produce gene trees. For this pangenome analysis we used panX (Ding et al., 2018) that takes GenBank files as input and extracts gene coordinates, annotations from GenBank and sequences from these genomes on both nucleotide and protein levels. For interactive visualization of pangenome clusters, alignments and phylogenetic trees we used panX visualization module obtain from github, https://github.com/neherlab/pan-genome-visualization/. Other databases showing greater detail about many viruses, such as Virus Pathogen Resource (VIPR, https://www.viprbrc.org) and GSAID (https://www.gisaid.org) are very useful but these resources do not provide gene clustering or pan-genome analysis.

### Re-annotation of Proteins From *Betacoronavirus* Genomes Using KAUST Metagenomic Analysis Platform (KMAP) Annotation Pipeline

To annotate uncharacterized genes in *Betacoronavirus*, we utilized all available protein sequences available from the clustering analysis as an input to our Automatic Annotation of Microbial Genomes (AAMG) pipeline (Alam et al., 2013), available via KAUST Metagenomic Analysis Platform (http://www.cbrc.kaust.edu.sa/kmap). AAMG uses sequence-based BLAST to UniProtKB (www.uniprot.org), KEGG (www.kegg.jp), and also Protein Family (PFam) domain detection using InterPro (www.ebi.ac.uk/interpro).

### Prediction of Function Using DeepGOPlus

We used all genes from *Betacoronavirus* dataset in order to explore Gene Ontology predictions through our in-house DeepGOPlus (Kulmanov and Hoehndorf, 2020) tool that combines deep convolutional neural network (CNN) model with sequence similarity-based predictions to derive relevant functional classes from Gene Ontology alongside a confidence score. Results were included with a score 0.1 and filtered for class specificity. Predicted Gene Ontology (GO) terms with a confidence score from DeepGOPlus were visualized using QuickGO (https://www.ebi.ac.uk/QuickGO/) for SARS-CoV-2 specific gene clusters. DeepGOPlus is available on github, https://github.com/bio-ontology-research-group/DeepGOPlus.

### Structure Based Prediction of Function

ProtParam (https://www.web.expasy.org/cgi-bin/protparam/protparam) was used for calculating protein features. Phobius (http://phobius.sbc.su.se/) and SignalP-5.0 were used for prediction of transmembrane regions and signal peptides (Almagro Armenteros et al., 2019). Jpred4 was used to calculate secondary structure features (http://www.compbio.dundee.ac.uk/jpred4/). 3D modeling was carried out using SwissModel (homology modeling) (Waterhouse et al., 2018) or QUARK (*ab initio* structure predictions) (Xu and Zhang, 2012).

### Mutation Detection in SARS-CoV-2 Isolates

We obtained high coverage isolate genomes for SARS-CoV-2 from http:/www.gisaid.org and filtered out any isolates which show any ambiguities such as stretches of Ns. Furthermore, we used Viral Annotation DefineR (VADR) from https://github.com/nawrockie/vadr to compare isolate sequences to reference SARS-CoV-2 model in order to further filter out any low quality isolates and to produce valid GenBank files. As a result, about 2,374 high quality isolates were obtained. GenBank files were further processed using panX (Ding et al., 2018) for clustering and alignment of nucleic acid and amino acid sequence of genes leading to detection of SNPs and phylogenetic analysis. Reduced alignments were used to produce a table of detected mutations and frequency, as shown in Supplementary Table 1. Interactive analysis of alignment of genes and phylogenetic trees for these isolates is available online at https://trackncov.cbrc.kaust.edu.sa.

## Results

**Figure 1:**
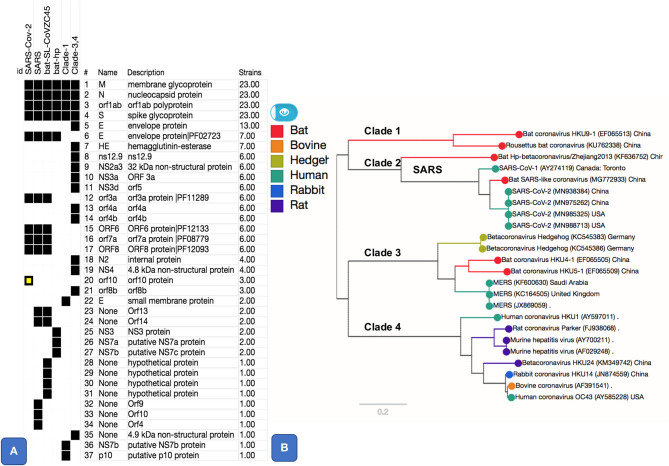
Pangenome core and accessory gene clusters based on sequence comparison of genes from genomes of genus *Betacoronavirus*. Core clusters appear in all genomes and accessory clusters appear in subset of genomes. Additional annotations such as Protein Family (PFAM) Ids, as shown for SARS-CoV-2 related clusters, were obtained using Automatic Annotation of Microbial Genomes (AAMG) pipeline (see methods). **(A)** Thirty-seven gene clusters are shown with a binary heatmap representing presence (black) or absence (white) of genes in *Betacoronavirus* clades 1, 2, 3, and 4. Clade 2 is expanded to show presence absence of genes for its members that include SARS-CoV-2, SARS, and two bat coronaviruses (MG772933 and KF636752). One of the gene cluster, orf10, marked in yellow, is a case of annotation artifact as it appears to be unique in SARS-CoV-2 according to annotations from GenBank, this gene is not predicted in any other genomes, however a TBLASTN search of this protein against NCBI's Nucleotide database (NT) show sequence matches this gene with 100% coverage in other SARS and SARS-like coronaviruses. **(B)** This panel shows a phylogenetic tree based on SNPs from core (M, N, orf1ab, and S) gene clusters. Tree is labeled with Clade numbers to distinguish SARS-like and other coronaviruses. Coloring of the tree is obtained based on related host information.

We used a pangenome approach to explore the genome of SARS-CoV-2 (MN985325 and related isolates) in comparison to other *Betacoronaviruses*, including SARS and MERS (see Table 1). Previous approaches (Wu et al., 2020) proceeded by extracting individual genes, aligning them, and then establishing a phylogeny. Our approach differs in that we first cluster all sequences to then calculate the alignment. This approach, based on using panX (Ding et al., 2018), allows us to achieve a higher sensibility in the detection of gene clusters. The resulting pangenome including all core and accessory gene clusters (Figure 1), interactive trees and alignments from 24 *Betacoronavirus* genomes, including 4 isolates from SARS-CoV-2, is available at https://pangenomedb.cbrc.kaust.edu.sa/.

### Essential Genes and Variation in Envelope Protein

**Figure 2:**
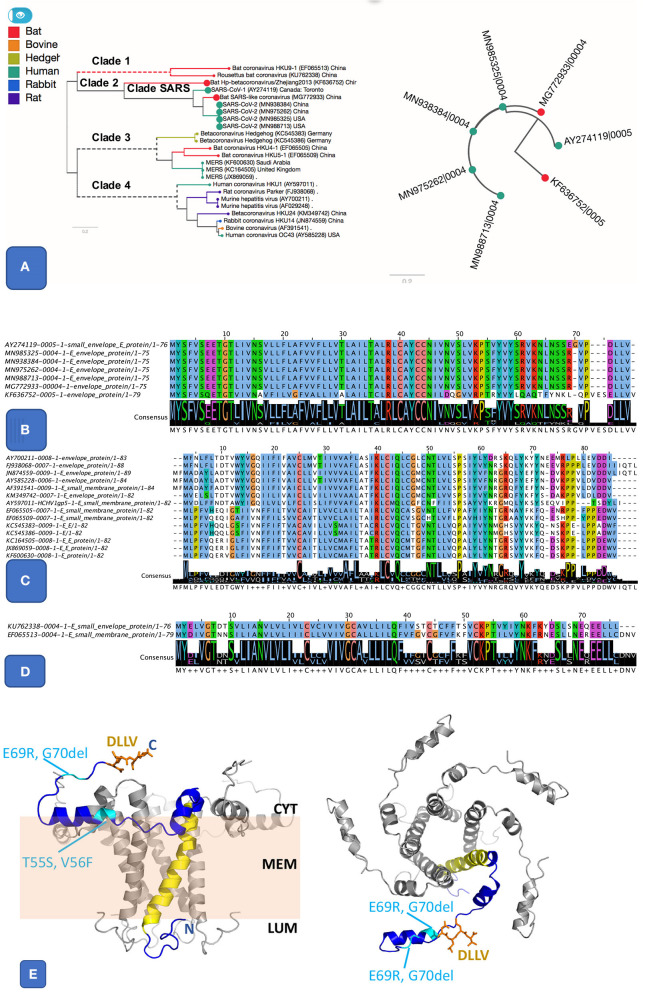
Pangenome analysis of 3 clusters related E protein. **(A)** The first E cluster, it shows much similar E proteins from SARS and SARS-like genomes, highlighted in the species tree (**A** left panel) alongside a gene tree (**A** right panel). **(B)**. Protein alignment of SARS and SARS-like E protein cluster. It includes SARS (AY274119), SARS-COV-2 (MN985325 and other isolates), and two bat coronaviruses (MG772933 and KF636752). The two important features are the ion-channel forming amino acids (where N15 and V25 were shown to be key for function) and the PBM class II motif (DLLV). Both features are completely conserved in SARS and SARS-CoV-2. **(C)** Protein alignment from another E cluster that groups E sequences from clade 3 and 4, including MERS and coronaviruses from other animals. **(D)** Protein alignment of 3rd E cluster that groups sequences from clade 1, related to two bat coronaviruses. **(E)** Theoretical model for the SARS-nCoV-1 protein E pentamer. *Left Panel*: side view. The membrane is illustrated in pale orange, and membrane (MEM), luminal side (LUM), and cytoplasmic side (CYT) are labeled. *Right panel*: view from the cytoplasm. One chain (blue) highlights protein features. N, C: location of N and C terminus, respectively. Yellow: IC; orange: DLLV (side chains are shown as stick models). Location of the residues changed in SARS-CoV-2 E are labeled and shown in cyan. The protein structure was modeled based on the NMR model PDB id 5 × 29, and completed using our in-house modeling program (to be published). The position of the region C-terminal to K53 was adjusted compared to the NMR model (see Supplementary Figure S6) to avoid its positioning entirely within the membrane, which appears unlikely given its amino acid composition (in particular R61, K63, and N64).

There are five essential genes in coronaviruses, the Spike protein (S), membrane glycoprotein (M), nucleocapsid (N), envelope protein (E), and the Orf1ab (a large polyprotein known as replicase/protease), all required to produce a structurally complete viral particle (Masters, 2006). These five genes define the core pan-genome. However, panX (Ding et al., 2018) only retrieved four of these (S, M, N, and the ORF1ab) as components of the core pan-genome (Figure 1). However, the fifth gene in the *Betacoronavirus* core genome, the envelope protein (E), varied among genomes, defining three distinct subclusters within the envelope protein E of *Betacoronaviruses* (Figure 2). One of these E clusters comprises only SARS (AY274119), SARS-CoV-2, and two Bat Coronaviruses (MG772933 and KF63652). Among the other two protein E gene clusters, one includes MERS (JX869059 and KC164505) and several coronaviruses from different animals, while the third cluster includes coronaviruses from two *Rousettus* bats. To validate the conservation and specificity of the SARS E cluster, we compared the SARS-CoV-2 E sequence with all known sequences in NCBI's non-redundant (NR) protein database. This comparison showed, consistent with our pan-genome analysis, that the subcluster of the protein E gene appearing in SARS-CoV-2, SARS and two bat coronaviruses, is defined by the same essential functional features, which are exactly conserved (100% identity) between bats and SARS-CoV-2, but differ slightly (95% identity with one deletion and three substitutions) from SARS (Figure 2B and Supplementary Information). A recent study (Wu et al., 2020) compared genomes and gene families of all alpha, beta, delta and gamma coronaviruses, but did not explore the variability present within the protein E gene sequences, which we did through the pangenome clustering approach reported above.

### Accessory Genes

We found five accessory gene clusters (15% of the accessory genome of genus *Betacoronavirus*) common to SARS-like coronaviruses, including SARS-CoV-2, out of 33 total accessory gene clusters. Four of these gene clusters (ORF3a, ORF6, ORF7a, and ORF8, Figure 1A), appear only in SARS-CoV-2, bat-SL-CoVZC45, and the SARS virus. A gene cluster annotated as ORF10 appeared to be unique to SARS-CoV-2 (Figure 1). However, a blast-based search against the DNA of all sequences in NCBI's NT database showed that the ORF10 sequence matches with 100% coverage in DNA of other SARS-like genomes, but there is no open-reading frame predicted for those genomes in the matching region (Figure 1A). Hence, the apparent uniqueness of ORF10 might simply be an artifact of the GenBank annotation pipeline that did not predict an ORF for this gene in other matching genomes. However, for completeness, we report a functional analysis of ORF10 as Supplementary Information since at present there is no experimental evidence available against or in favor of this being a real gene.

### Annotation of Uncharacterized Genes

Compared to the essential genes S, M, N, E, and ORF1ab, other genes such as ORF3a and ORF7a are poorly characterized, according to GenBank annotations (https://www.ncbi.nlm.nih.gov/genome/proteins/86693?genome_assembly_id=760344). Our bioinformatic protein structural analysis confirmed that SARS-CoV-2 ORF3a (cluster 12), also known as viroporin, and ORF7a (cluster 17), that is same as ORF8a (Castano-Rodriguez et al., 2018) in SARS, retain the structural features observed in other SARS viruses, namely a multi-pass transmembrane domain and a cytoplasmic β-barrel or β-sandwich fold (ORF3a) and an N-terminal *sec*-pathway signal peptide, cleaved after residue 15, an immunoglobulin-like β-sandwich fold stabilized by two cysteine di-sulfide bonds, and a C-terminal single-pass transmembrane helix (ORF7a; Supplementary Figures S5, S6). Hence these SARS-CoV-2 proteins are expected to act in the same way as they do in other SARS, namely as accessory proteins mostly localized in the endoplasmic reticulum-Golgi intermediate compartment, but also occurring on the cell membrane where they enhance viral pathogenicity and mortality through protein-protein interactions (Hanel et al., 2006; Minakshi et al., 2009; DeDiego et al., 2014).

The remaining two accessory gene clusters, ORF6, ORF8, present in SARS-CoV-2, SARS, and bat-SL-CoVZC45 are functionally uncharacterized. All are very short polypeptides (61, 121 and 38 residues for ORF6 and 8, respectively) with a large percentage of hydrophobic residues (62 and 56%, respectively). None of the proteins have trans-membrane regions, but ORF8 has an N-terminal *sec*-pathway signal peptide with a cleavage site after residues 15, suggesting that it is secreted into the extracellular space (Supplementary Figures S5, S6). Following signal peptide cleavage, the ORF8 protein core is predicted to consist largely of β-strands and features 7 cysteines. We predict that this protein adopts a cysteine disulfide-bond stabilized β-sandwich structure similar to the soluble domain of ORF7a (Supplementary Figures S5, S6), inferring that ORF8 also functions as ligand binding module. ORF6 consists of a long amphipathic helical region, followed by an acidic tail. Our deep-learning-based annotation [DeepGOPlus Kulmanov and Hoehndorf, 2020] suggested that ORFs 6, 7, 8 are involved in the regulation of molecular functions, either in response to stimuli (ORFs 8), or in cellular component biogenesis and organization (ORF6) (Supplementary Figures S2–S4). Collectively, our analysis suggested no major function or gene cluster to be unique to SARS-CoV-2. Moreover, our analysis is in agreement with ORFs 3a, 6, 7a, 8, and being accessory non-essential proteins, that would be inefficient targets for COVID-2019 therapy.

### Mutations in SARS-CoV-2 Isolates

**Figure 3:**
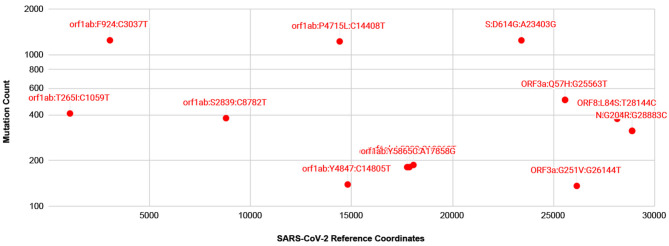
Prevalent mutations detected from comparing over 2,000 high quality SARS-CoV-2 isolates. Mutations are labeled here as gene name followed by potential amino-acid mutation and nucleotide mutation, joined by colon character, “:”. Majority of prevalent mutations were detected in gene orf1ab. Among non-synonymous mutations two mutations, one in orf1ab and one in Spike protein, S, appear in over 1,000 isolates. A full list of mutations detected are shown in Supplementary Table 1.

A number of isolates from COVID-19 patients are now being made available from several countries, thanks to resources such as gisaid.org. For mutation analysis, from over 10,000 SARS-CoV-isolates available at gisaid.org, we processed around 2,000 isolates with no ambiguous bases. The frequency of mutations detected, so far, is shown in Figure 3. There are thirteen prevalent mutations confirmed in a minimum of 100 isolates. Among these, there is one synonymous and two non-synonymous mutations appearing in over 1,000 isolates, one in Spike protein and two in gene orf1ab. A full list of ~529 mutations detected with a frequency confirmed by minimum two isolates is presented in Supplementary Table 1. There are a few low frequency <5 isolates mutations detected in Spike protein Receptor Binding Domain (RBD) but these do not interact with human ACE2 (Ortega et al., 2020). Moreover, no mutations are detected in Ion Channel forming region of E protein. Alignments from isolates analyzed are available at https://trackncov.cbrc.kaust.edu.sa.

## Discussion

The S protein binds to the host receptor ACE2, and hence presents a prime target for preventing viral entry into host cells (Li, 2016). The S proteins from SARS and SARS-CoV-2 are similar (Wan et al., 2020), but structural differences in the receptor binding domain (RBD) preclude the use of SARS–specific neutralizing antibodies to inhibit SARS-CoV-2 (Wrapp et al., 2020). We therefore focused our attention on the protein E, which is highly similar in SARS-CoVs. The E protein, also a viroporin (Liao et al., 2006), was previously confirmed as a determinant of pathogenicity (Jimenez-Guardeno et al., 2014; Nieto-Torres et al., 2014) in SARS. Protein E was used as target for SARS antivirals (Torres et al., 2015), and studies using SARS-CoV with lacking or mutated protein E as vaccine candidates showed promising results (DeDiego et al., 2007; Lamirande et al., 2008; Regla-Nava et al., 2015; Schoeman and Fielding, 2019). Furthermore, the E gene is one of the key genes used in identification of SARS group, however combination of probes from additional genes such as RdRP and N can unambiguously help identify SARS-CoV-2 (Corman et al., 2020; Huang et al., 2020).

The SARS protein E has ion channel (IC) activity and features a post-synaptic density-95/discs large/zona occludens-1 (PDZ)-binding motif (PBM). Both IC and PBM were required for SARS-CoV to induce virulence in mice (Castano-Rodriguez et al., 2018). The protein E from SARS-CoV-2 differs from that of SARS only by three substitutions and one deletion (Figures 2A,B). The PBM and IC are identical in all SARS E proteins in SARS subcluster, but distinct in orthologs from the other two E subclusters (Figures 2B–D). The substitutions and insertions are positioned in flexible cytoplasmic regions, where they are not expected to affect the protein structure, IC or function of the PBM (Figure 2E).

Studies on SARS demonstrate that the E protein uses the IC and PBM to trigger a cytokine storm that activates the inflammasome, leading to increased edema in lungs. Ultimately these events result in ARDS (Jimenez-Guardeno et al., 2014; Nieto-Torres et al., 2014; Torres et al., 2015), one of the leading causes of death in SARS and SARS-CoV-2 infection (Huang et al., 2020; Xu et al., 2020). The high level of conservation of the IC and PBM within clade 2, specifically SARS and SARS-CoV-2, strongly suggests that E protein of SARS-CoV-2 may be a target for therapy that can possibly be inhibited with drugs already tested on SARS. For example, the drugs AMT (1 amino-adamantane) or more effectively HMA (5-N,N-Hexamethylene amiloride) have been shown to block the IC activity of SARS-CoV-1 (Wilson et al., 2006; Behmard et al., 2018) and restrict its reproduction, leading to better survival of the animal host. Another compound BIT225 also appear to be a potent inhibitor of IC activity in p7 viroporins (Behmard et al., 2018), see reference (Jalily et al., 2020) for more options. From the natural compounds, Glycyrrhizin, the root extract of *Glycyrrhiza glabra* (liquorice) has been shown previously that its high dose can completely block the replication of SARS virus (Cinatl et al., 2003). Recent docking experiments show Glycyrrhizin (doi: 10.26434/chemrxiv.12286421.v1) as well as some other phytochemicals (Gupta et al., 2020) have the potential to block E protein IC. A more recent study (Xia et al., 2020) reported *in vitro* and *in vivo* results for existing and a few new E protein inhibitors showing potent anti-SARS-CoV-2 activity.

As numerous compounds were shown previously to interact with SARS-CoV E protein (Pervushin et al., 2009). Given the conservation of the key features of E proteins from SARS anSARS-CoV-2, these compounds would be promising candidates for inhibiting ARDS in COVID-19. Some of the compounds were found to interact directly with the residues placed inside the channel (Pervushin et al., 2009; Surya et al., 2018), analogous to a well-established mechanism for the tetrameric M2 channel. It seems the mechanisms of action vary among the various viroporins, like the amantadine bound to hexameric p7 channel occupying the hydrophobic clefts in a 6:1 amantadine:hexamer ratio (OuYang et al., 2013; Oestringer et al., 2018). Nevertheless, an amantadine structure in a complex with viroporin channel was determined to interact with tetrameric M2 and a *de novo* designed trimeric protein in a 1:1 drug:oligomer stoichiometric ratio (Stouffer et al., 2008; Park et al., 2019). The hexameric p7 protein channel formed a 1:6 ratio channel to drug complex, although, there was a substantial impact on p7 oligomeric state of protein to detergent ratio affecting the amantadine affinities to p7 polypeptide was detected (Oestringer et al., 2018; Chen et al., 2019). In addition, channels are believed to exist in at least two states, open and closed, as evidenced by conductivity experiments (Nieva et al., 2012). One common mechanism of drugs blocking the ion conductance is closing the passage through the formed oligomeric tunnel, likely through 1:1 drug:channel ratio, although other ratios were observed too, as with SARS-CoV E-protein and HMA (Pervushin et al., 2009). Thus, one can assume that drugs eliminate the channel functionality by forming a stable, more inert complex with functional viroporin channels, or at least, drugs inhibit intermediate step as functional channels form. We learned that M2 mutations leading to resistance against amantadine and its analogs are caused by substitutions of amino acids in the TM segment (Jalily et al., 2020). This changes the hydrophobic volume and the geometry of the channels while still preserving their main function, ion conductivity. In the case of E protein in SARS-CoV-2, an analysis of mutations detected so far from over 2,000 isolates show that at present there are no prevalent or low frequency mutations detected in the TM or Ion channel forming residues (Figure 3 and Supplementary Table 1).

Additionally, the PBM enables the E protein to interact with the cellular PDZ domains to evade host immune system (Gutierrez-Gonzalez and Santos-Mendoza, 2019), and might also a good target. However, there are no specific high-affinity compounds that would inhibit this protein-protein interaction motif. Its association with the tandem PDZ domains of syntenin activates the MAP kinase p38, triggering the overexpression of inflammatory cytokines. Consequently, inhibition of p38 by SB203580 increases the survival of the host (Jimenez-Guardeno et al., 2014). Such more indirect targeting of the protein E function might also be considered for COVID-19 therapy.

## Conclusions

Therapies to combat the spread of SARS-CoV-2 and the lethality caused by the resulting COVID-2019 are currently focusing primarily on S, the viral spike protein (Kruse, 2020; Liu et al., 2020; Wrapp et al., 2020). However, despite high similarities between proteins S between SARS and SARS-CoV-2 viruses, existing neutralizing antibodies are ineffective against SARS-CoV-2(Wrapp et al., 2020). Hence, new antibodies that bind specifically to the SARS-CoV-2 spike protein need to be developed, tested, and approved for human use, which would be a time-consuming process. Our pangenomic analysis suggests that the protein E of all SARS viruses preserves its critical motifs used for pathogenesis, and should be considered as an alternative target to be tested for therapies to mitigate COVID-2019.

## Data Availability Statement

All datasets presented in this study are included in the article/Supplementary Material and https://pangenomedb.cbrc.kaust.edu.sa.

## Author Contributions

IA, TG, and CD conceived and designed the research. IA led the study and conducted the data analysis. AK helped and developed the web-based resource and led computational components of the study. ŁJ, MK, and SA contributed to functional and structural analysis. AP contributed with inferences on virulence. IA and CD developed the first draft of the manuscript. All authors contributed to writing and improving the manuscript and approved the submission.

## Conflict of Interest

The authors declare that the research was conducted in the absence of any commercial or financial relationships that could be construed as a potential conflict of interest.
