# Supplementary material for: Functional Pangenome Analysis Shows Key Features of E Protein Are Preserved in SARS and SARS-CoV-2
Source: Front Cell Infect Microbiol. 2020 Jul 27;10:405. doi: 10.3389/fcimb.2020.00405 (PMC7396417; doi:10.3389/fcimb.2020.00405)
Supplement: Supplementary file 1 [file Data_Sheet_1.pdf]

Supplementary Materials

Supplementary Information, ORF10 gene in SARS-CoV-2:

A short gene predicted in in SARS-CoV-2, known as ORF10, appears unique only to SARS-CoV-2. A sequence comparison of this gene to DNA of all *Betacoronavirus* genomes in NCBI shows matches in SARS and SARS-like genomes with 100% coverage, although no gene has been predicted in matching location of ORF10 in SARS or SARS-like genomes. This raises a question whether ORF10 is an artifact of annotation in SARS-CoV-2 where it has been predicted or an artifact in SARS and SARS-like genomes where it has not been predicted. There is no experimental data available to approve or disprove the reality of this gene. For completeness purposes we included ORF10 in our functional and structural analysis of SARS-CoV-2 but not included in the main text.

Supplementary Figure 4-6 shows predicted Gene Ontology and structural information about ORF10 using deep learning Gene Ontology Prediction tool (DeepGOplus). It shows that, similar to ORF8, this gene regulates molecular functions and contributes to response to stimulus. It is predicted to be localized only in the extracellular region of the host cells. ORF10 is predicted to harbor a long helix followed by a β-strand and appears to be localized only in the extracellular region of the host cells. It has been concluded that SARS-CoV-2 accessory genes, including ORF10, carry a helper function and do not serve as a major target for detection or therapy of COVID-2019.

Supplementary Figures

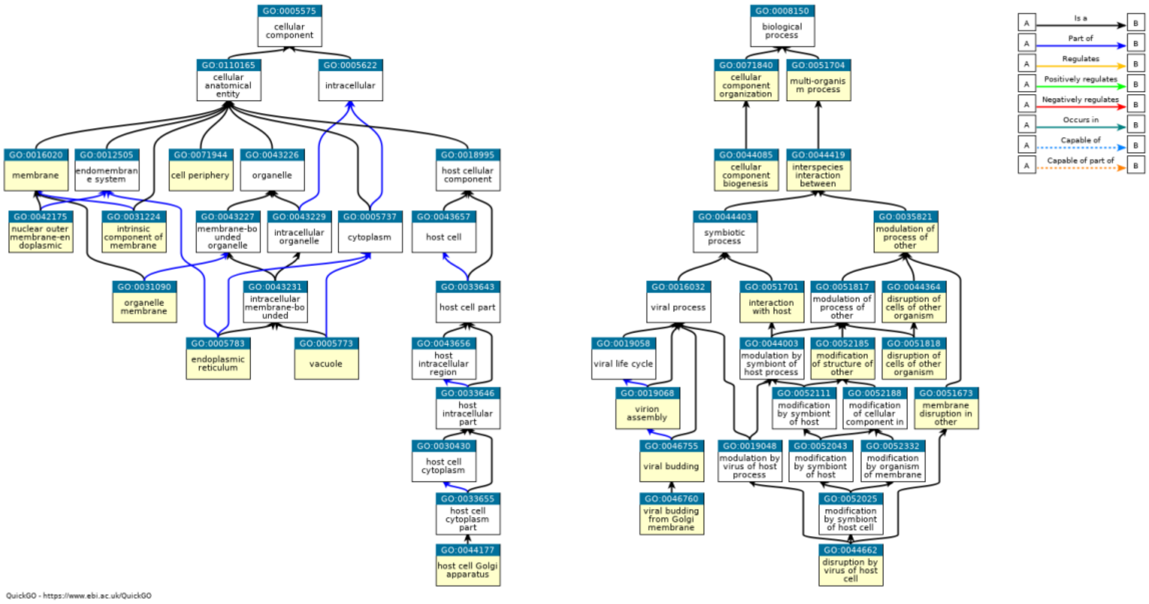

*Supplementary Figure S*1.

DeepGOPlus predicted Gene Ontology Graph for SARS-CoV-2 envelope protein, E.

According to predictions, the protein can be localized in the host cell Golgi apparatus, vacuoles, endoplasmic reticulum, and membrane cell periphery of the host cell. It contributes to the process of disruption of the host cell and viral budding from Golgi membrane.

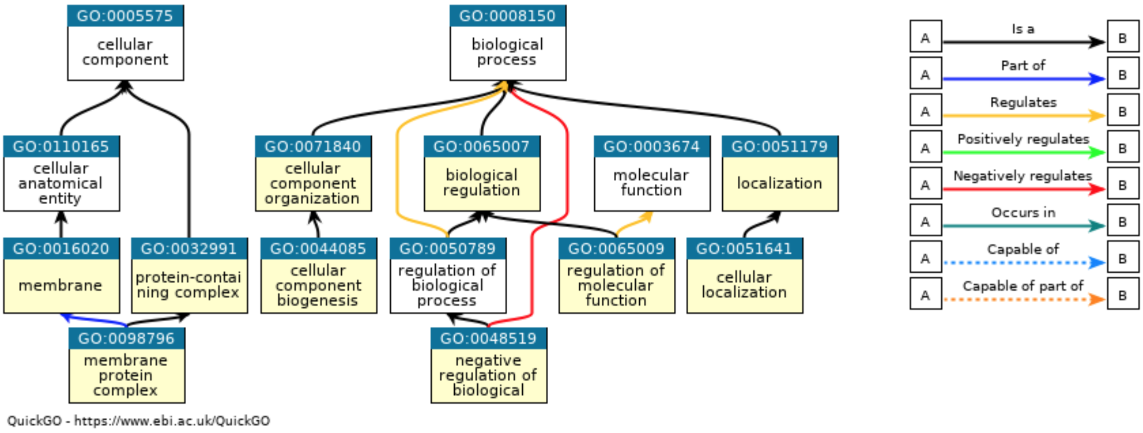

Supplementary Figure S2.

DeepGOPlus predicted Gene Ontology Graph for SARS-CoV-2 ORF6. Predictions generally describe this protein to be localized in a membrane protein complex. It negatively regulates biological processes and contributes to cellular localization and biogenesis.

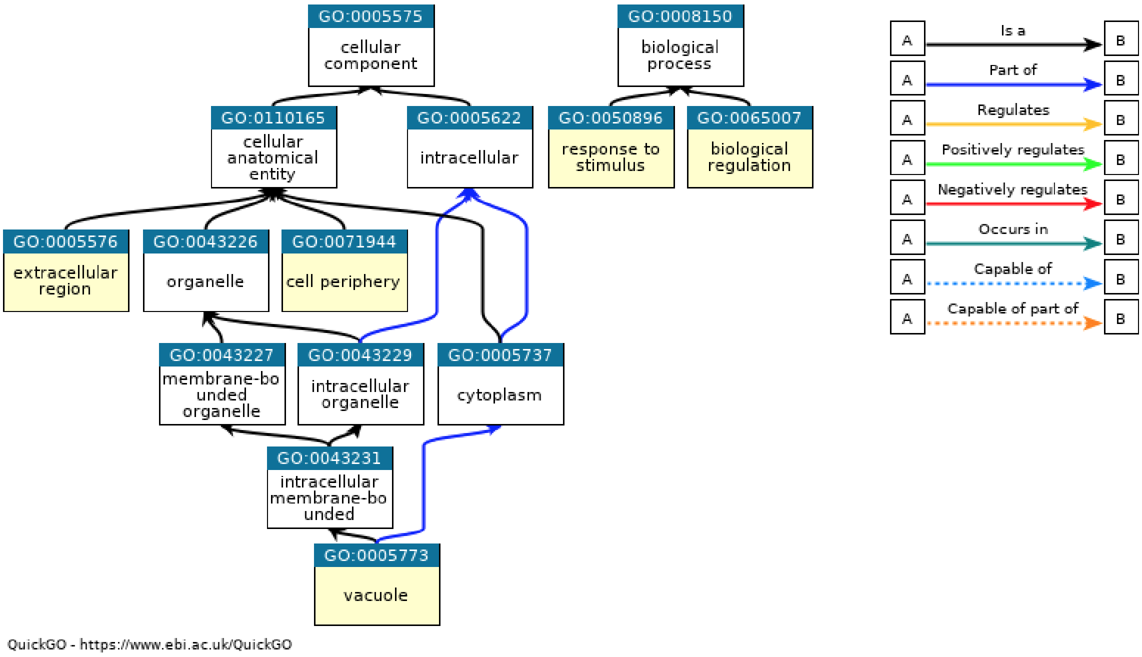

Supplementary Figure S3.

DeepGOPlus predicted Gene Ontology Graph for SARS-CoV-2 ORF8. This protein contributes to processes as response to stimulus along with biological

regulation and potentially localized in vacuoles, cell periphery and extracellular regions.

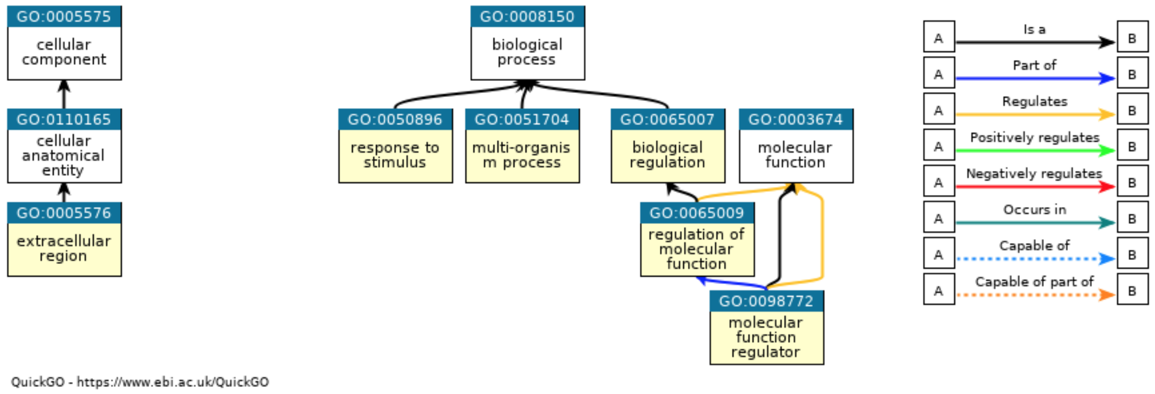

Supplementary Figure S4.

DeepGOPlus Gene Ontology Graph for SARS-CoV-2 gene, ORF10. Similar to ORF8, this protein (if this predicted ORF is not an annotation artifact, see Figure 1) regulates molecular functions and contributes to response to stimulus. It is predicted to be localized only in the extracellular region of the host cells.

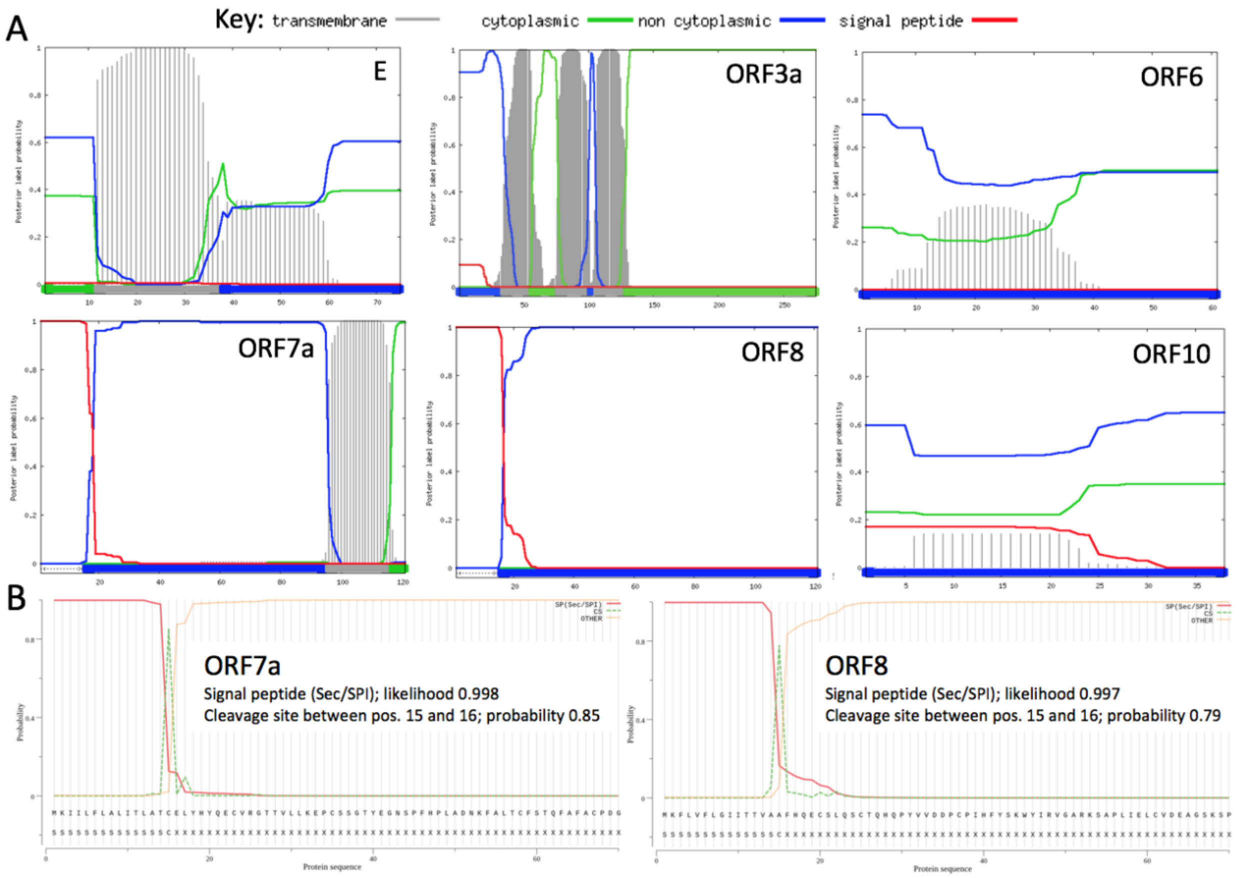

Supplementary Figure S5

Prediction of signal peptides and transmembrane regions. A) combined prediction of signal peptides and transmembrane regions, and (B) an additional analysis to identify the type of signal peptide and cleavage site for ORF7a and ORF10.

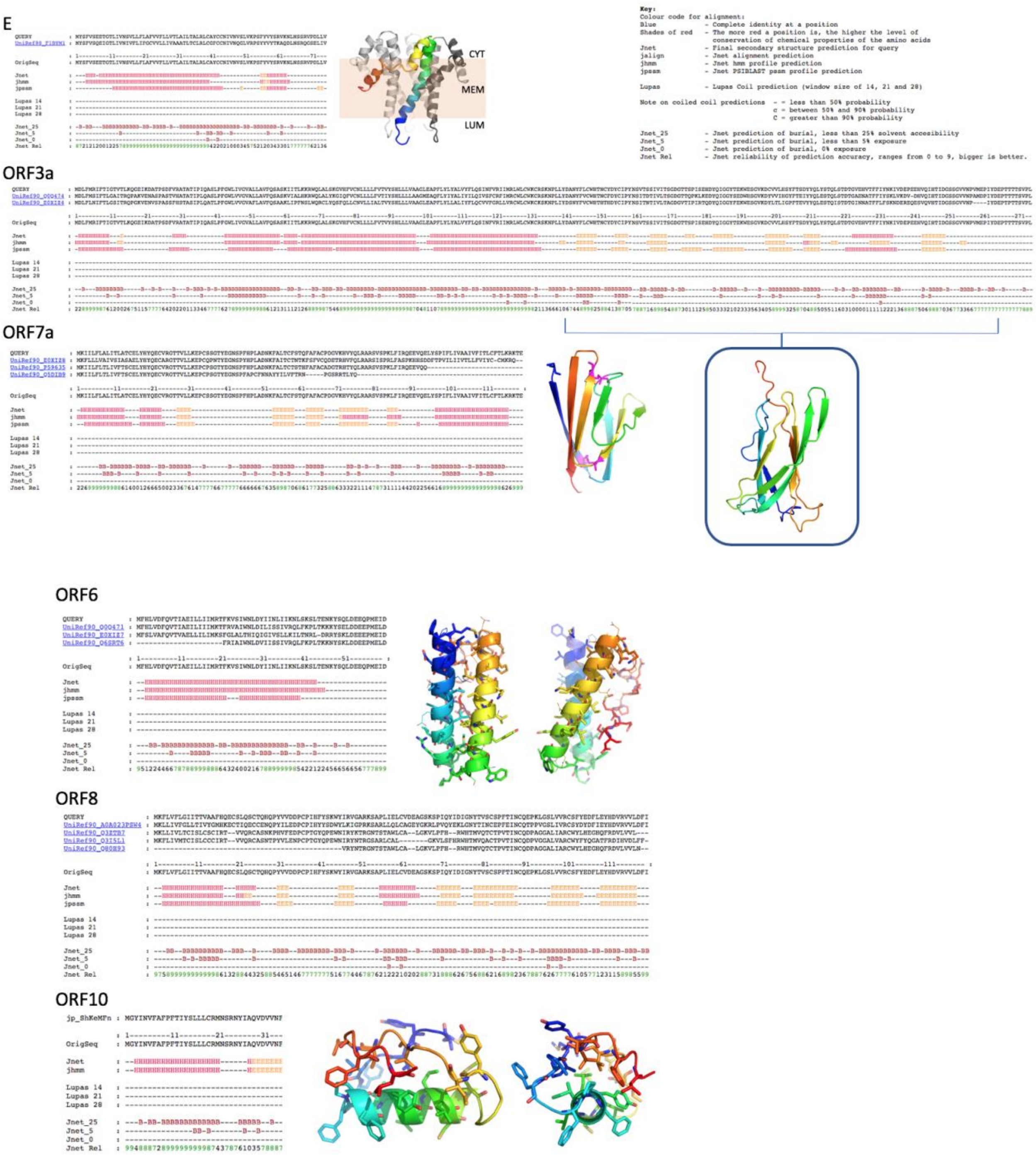

Supplementary Figure S6

Prediction of secondary structure characteristics and tertiary architecture (colour-ramped from N- (blue) to C-terminus (red)). E: homology model based on the pentameric SARS CoV E protein (PDB Id 5x29, 26% sequence identity). Only one

chain is colour-ramped. The membrane (pale pink) is indicated with cytoplasmic and luminal sides labelled. Note that the N-terminal 7 and C-terminal 15 residues are not included in the model. ORF3a and ORF7a: structural models for the soluble domains. The two di-sulphide bridges conserved in SARS ORF3a are shown in magenta (template: 1xak). 90˚ views of *ab initio* models are shown for ORF6 and ORF10. Hydrophobic and charged side chains are highlighted in ORF6, and hydrophobic side chains are highlighted in ORF10.

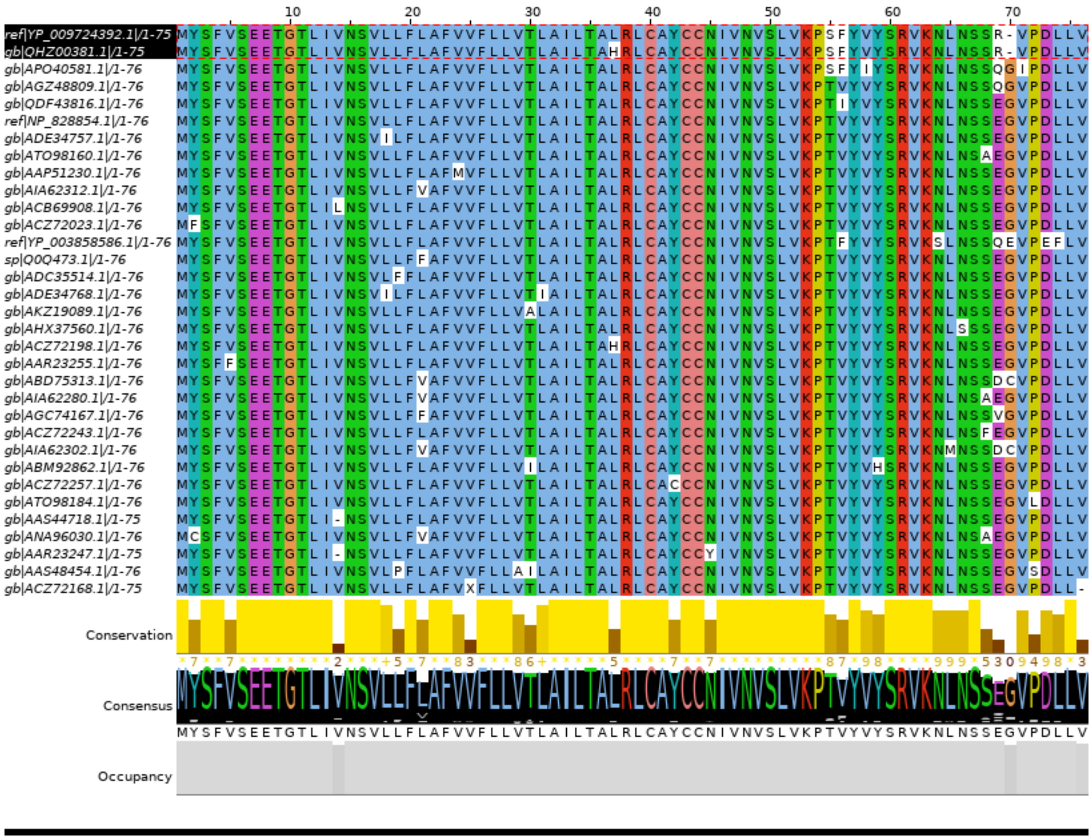

Supplementary Figure S7. Alignment of SARS-CoV-2 E protein comparison to hits available in NCBI's NR database. The first two appear from SARS-CoV-2 and rest of the hits appear from SARS and SARS-like *Betacoronaviruses.* This result shows SARS-CoV-2 E protein is conserved only in SARS clade and E from MERS or other animal coronaviruses clusters in different clusters (see Figure 2).
